# miR-218-5p/RUNX2 Axis Positively Regulates Proliferation and Is Associated with Poor Prognosis in Cervical Cancer

**DOI:** 10.3390/ijms23136993

**Published:** 2022-06-23

**Authors:** Merlin Itsel Cruz-De la Rosa, Hilda Jiménez-Wences, Judit Alarcón-Millán, Manuel Joaquín Romero-López, Carlos Alberto Castañón-Sánchez, Eric Genaro Salmerón-Bárcenas, Gloria Fernández-Tilapa

**Affiliations:** 1 Laboratorio de Investigación Clínica, Facultad de Ciencias Químico Biológicas, Universidad Autónoma de Guerrero, Avenida Lázaro Cárdenas S/N, Ciudad Universitaria Sur, Col. La Haciendita, Chilpancingo, Guerrero 39087, Mexico; itsel_cruz@hotmail.com (M.I.C.-D.l.R.); wences2009@hotmail.com (H.J.-W.); juditm@uagro.mx (J.A.-M.); mjrl_0502@hotmail.com (M.J.R.-L.); 2 Laboratorio de Investigación en Biomoléculas, Facultad de Ciencias Químico Biológicas, Universidad Autónoma de Guerrero, Avenida Lázaro Cárdenas S/N, Ciudad Universitaria Sur, Col. La Haciendita, Chilpancingo, Guerrero 39087, Mexico; 3 Laboratorio de Investigación Biomédica, Hospital Regional de Alta Especialidad de Oaxaca, San Bartolo Coyotepec, Oaxaca 71256, Mexico; 4 Departamento de Biomedicina Molecular, Centro de Investigación y de Estudios Avanzados del Instituto Politécnico Nacional, Mexico City, Ciudad de México 07360, Mexico; egsb.1990@gmail.com

**Keywords:** cervical cancer, miR-218-5p, *RUNX2*, C-33A, CaSki

## Abstract

The overexpression of miR-218-5p in cervical cancer (CC) cell lines decreases migration, invasion and proliferation. The objective was to identify target genes of miR-218-5p and the signaling pathways and cellular processes that they regulate. The relationship between the expression of miR-218-5p and RUNX2 and overall survival in CC as well as the effect of the exogenous overexpression of miR-218-5p on the level of RUNX2 were analyzed. The target gene prediction of miR-218-5p was performed in TargetScan, miRTarBase and miRDB. Predicted target genes were subjected to gene ontology (GO) and pathway enrichment analysis using the Kyoto Encyclopaedia of Genes and Genomes (KEGG). The miR-218-5p mimetic was transfected into C-33A and CaSki cells, and the miR-218-5p and RUNX2 levels were determined by RT–qPCR. Of the 118 predicted targets for miR-218-5p, 86 are involved in protein binding, and 10, including RUNX2, are involved in the upregulation of proliferation. Low miR-218-5p expression and a high level of RUNX2 are related to poor prognosis in CC. miR-218-5p overexpression is related to decreased RUNX2 expression in C-33A and CaSki cells. miR-218-5p may regulate RUNX2, and both molecules may be prognostic markers in CC.

## 1. Introduction

Cervical cancer (CC) is one of the leading causes of cancer-related death in women. Worldwide, CC ranks fourth in incidence and mortality, with 604,127 new cases and 341,831 deaths per year. Overall, 78% of cases of CC are diagnosed in women between 30 and 39 years old, 21% in women from 20 to 29 years old, and the 1% in women under 20 years old. In developing countries, 452 new cases of CC were reported, and CC occupied second place behind malignant neoplasms in female patients [1,2,3]. Factors that contribute to the carcinogenesis of the uterine cervix are smoking, the use of oral contraceptives [2,4], infection with high-risk human papillomavirus (HR-HPV), and genetic and epigenetic factors that play a fundamental role in malignant transformation [4,5].

The deregulation of the expression of microRNAs (miRNAs) is among the epigenetic alterations that characterize the various stages of cervical carcinogenesis. Evidence indicates that more than 281 miRNAs with oncogenic or tumor suppressor functions [6] are involved in the development of CC [7,8]. miR-218-5p has been found to be decreased in CC samples and cell lines, and is considered a tumor suppressor in this malignancy [9,10,11,12,13]. In the cervical tissue of women with CC and with a high-grade squamous intraepithelial lesion (HSIL) or grade II/III cervical intraepithelial neoplasia (CIN), the expression of miR-218-5p is significantly decreased compared to that in a low-grade squamous intraepithelial lesion (LSIL) or CINI and that in normal tissue [10,11,12,13,14,15,16,17]. Decreased expression of miR-218-5p is associated with metastasis to lymph nodes, tumor size, degree of tumor differentiation and spread to blood vessels [13,14,15,16,17,18]. miR-218-5p expression negatively correlates with Ki67 in CC tissues [14].

The overexpression of miR-218-5p in the CC, CaSki, ME180, HeLa and Yumoto cell lines is related to decreased migration and invasion and deregulation of the expression of oncogenes, including *LAMB3.* The LAMB3 protein is a laminin that participates in the regulation of cell proliferation, migration and adhesion [10]. In HeLa cells transfected with miR-218-5p mimics, the expression of the oncogenes *IDO1*, *HMGB1* and *RAGE* is inhibited, cell viability is reduced, and apoptosis is increased [12,18]. On the other hand, in SiHa and ME-180 cells, the overexpression of miR-218-5p significantly reduced the level of survivin, a direct target of this miRNA. Survivin is a small protein that regulates mitosis and inhibits caspase-dependent apoptosis. Experimentally, it was confirmed that the miR-218-5p–survivin axis regulates the clonogenicity, migration and invasion of CC cell lines [19]. In SiHa cells transfected with a siRNA against E6 of HPV16, the expression of miR-218-5p and the level of E-cadherin increased, the expression of N-cadherin decreased, the epithelial–mesenchymal transition (EMT) was reversed, and the invasive capacity of cells was determined. In CC, experimental evidence indicates that miR-218-5p inhibits EMT and cell invasion through the regulation of SFMBT1 and DCUN1D1 mRNAs, proteins that promote metastasis [20]. On the other hand, the overexpression of miR-218-5p increases the rate of apoptosis and decreases the proliferation of SiHa cells through the regulation of GLI3 [14]. Clonogenicity assays showed that in SiHa, HeLa, C-33A and CaSki cells, the loss of miR-218-5p can predict radioresistance, and its increase improves radiosensitivity and significantly increases radiation-induced apoptosis [15]. The published information suggests that, in CC, miR-218-5p can be a therapeutic target or a prognostic factor; however, much remains to be discovered about the target genes, the molecular mechanisms and the cellular processes regulated by this miRNA.

Runt-related transcription factor 2 (RUNX2) is a transcription factor (TF) that belongs to the TF family RUNX, which includes two other members, RUNX1 and RUNX3. The TF proteins in this family contain a DNA-binding subunit called the Runt domain, which consists of 128 amino acids [21,22]. RUNX2 regulates cellular processes such as proliferation and the cell cycle during embryonic development. In addition, it is involved in skeletal growth [23] and breast and thyroid morphogenesis [24]. It has been reported that RUNX2 is overexpressed in different tumors derived from epithelial tissues, such as breast [25], pancreas [26], prostate [27], and lung [28], among others [29]. RUNX2 induces the expression of various genes involved in tumor progression, including *VEGF*, *MMP9*, *OPN*, *SNAI 1* and *2*, *TWIST1* and *TIMP13* [29,30], and activates signaling pathways such as mTORC2, PTEN/PI3K/AKT and NF-κB [30,31,32]. RUNX2 is overexpressed and positively regulates the invasion of SiHa and C-33A cells [33].

Based on the published data on miR-218-5p and RUNX2, the level of RUNX2 has not been reported in tissues of patients with CC, and it is unknown whether the expression of RUNX2 is related to the deregulation of miR-218-5p in CC. The objective of this study was to identify target genes of miR-218-5p and the signaling pathways and cellular processes in which these genes participate, and to evaluate whether some pathways or processes are among those that contribute to CC progression. Additionally, we aimed to analyze the expression of miR-218-5p and RUNX2 from the data recorded in The Cancer Genome Atlas (TCGA) and to verify their relationship with patient survival. Finally, we explored the effect of miR-218-5p overexpression on RUNX2 mRNA levels in C-33A and CaSki cells.

Through bioinformatic analysis, it was found that RUNX2 is a potential target of miR-218-5p. The data recorded in TCGA indicate that RUNX2 is overexpressed and miR-218-5p levels are low in CC tissues. The high level of RUNX2 expression and the decrease in miR-218-5p are related to the low survival of patients with CC. The overexpression of miR-218-5p is related to a decrease in RUNX2 expression in C-33A and CaSki cells.

## 2. Results

### 2.1. Low miR-218-5p Expression Is Associated with Poor Prognosis in Patients with CC

**Figure 1:**
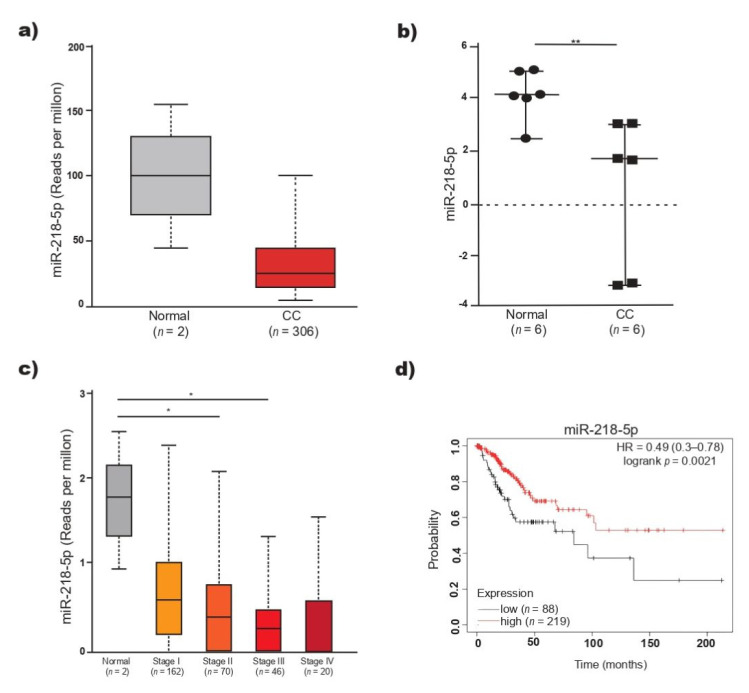
Expression and prognostic value of miR-218-5p in CC. (**a**) miR-218-5p expression in CC and normal tissues (TCGA data), (**b**) miR-218-5p expression in the GSE86100 dataset, including samples of squamous cell carcinoma positive for HPV16 and normal tissues (healthy uninfected cervix), (**c**) miR-218-5p level for tumor stage in CC, based on FIGO criteria, (**d**) Kaplan–Meier survival curve, the red line represents the patients with high expression of miR-218-5p and the black line represents the patients with low expression of miR-218-5p. miR-218-5p expression was significantly related with overall survival (OS) of CC patients (TCGA data). * *p <* 0.05, ** *p* < 0.01.

The expression level of miR-218-5p was analyzed by means of data from 306 patients with CC recorded in TCGA. miR-218-5p was found to be decreased in CC (*n* = 306) compared to normal tissue (*n* = 2), but the differences were not statistically significant (Figure 1a). Considering that the result may be influenced by the low number of normal tissue samples, the expression of miR-218-5p was analyzed in the GSE86100 dataset. The levels of miR-218-5p were significantly lower in CC than in normal tissue (*p* < 0.05) (Figure 1b) and lower in cancer tissue samples than in normal tissue samples analyzed by sequencing and microarrays. The level of miR-218-5p in 298 CC samples recorded in TCGA was analyzed in relation to the CC stage. The expression of miR-218-5p gradually decreased as the tumor stage increased (FIGO stage I to IV). In stage III, the level of miR-218-5p was significantly lower than that in normal tissue (Figure 1c). OS analysis was performed to estimate the prognostic value of miR-218-5p. The KM estimator revealed a significantly reduced OS in patients with low miR-218-5p expression (HR = 0.49, 95% CI = 0.3–0.78, *p* < 0.05) (Figure 1d). A low level of miR-218-5p in CC tissues was associated with a poor patient prognosis.

### 2.2. miR-218-5p Regulates Genes Involved in the Positive Regulation of Cell Proliferation

**Figure 2:**
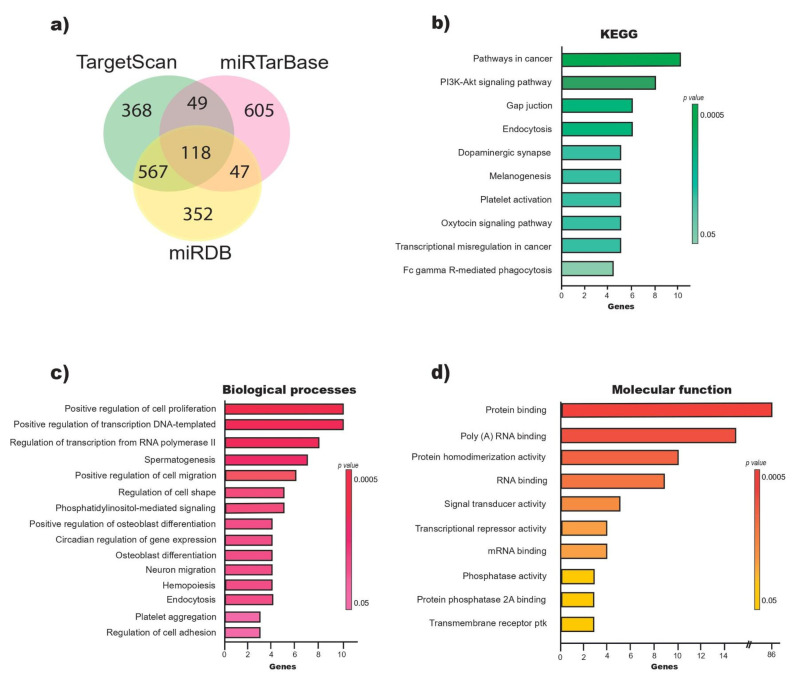
Prediction of miR-218-5p target genes and functional analysis (Top of pathways). (**a**) Number of miR-218-5p target genes predicted in TargetScan, miRTarBase and miRDB. (**b**) KEGG analysis of 118 miR-218-5p target genes overlap in the three databases. (**c**) Biological processes in the participate the 118 miR-218-5p target genes and (**d**) molecular function.

The target prediction analysis of miR-218-5p suggested 1102 genes in TargetScan, 1084 in miRDB and 819 in miRTarBase. A total of 118 overlapping genes were considered probable targets of miR-218-5p (Figure 2a, Table 1) and were subjected to GO and KEGG analysis. The results show that 15.3% (18/118) of the genes were significantly linked to cancer-related pathways and the PI3K–AKT signaling pathway (Figure 2b). The GO analysis showed significant enrichment of the following biological processes (BP) by the target genes of miR-218-5p: positive regulation of cell proliferation (*p =* 0.002), positive regulation of transcription, DNA-templated (*p =* 0.004), and positive regulation of migration (*p* = 0.005) (Figure 2c). On the other hand, the target genes of miR-218-5p are involved in molecular functions (MF) such as protein binding, binding to polyadenylated RNA and protein homodimerization (Figure 2d). The target genes of miR-218-5p involved in positive regulation of proliferation include *PDGFRA*, *CUL3*, *KIT*, *NACC1*, *RARA*, *BIRC6*, *GAB2*, *CACUL1*, *GNAI2* and *RUNX2* (Table 1).

### 2.3. RUNX2 Overexpression Correlates with Poor Overall Survival in CC

**Figure 3:**
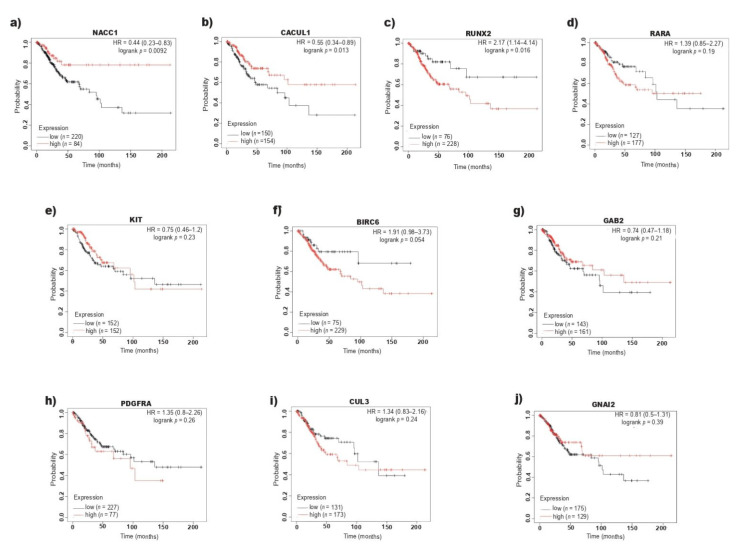
Effects of expression of miR-218-5p target genes on the survival of patients with CC. (**a**–**j**) Analysis of prognostic value of miR-218-5p target genes involved in the positive regulation of cell proliferation in cervical tissue of CC patients. (**a**) NACC1, (**b**) CACUL1, (**c**) RUNX2, (**d**) RARA, (**e**) KIT, (**f**) BIRC6, (**g**) GAB2, (**h**) PDGFRA, (**i**) CUL3, (**j**) GNAI2. The Kaplan–Meier survival curves show the prognostic importance for NACC1, CACUL1 and RUNX2. (*p* < 0.05). The red line represents the patients with high expression and the black line represents the patients with low expression of miR-218-5p target genes.

The target genes of miR-218-5p involved in the positive regulation of cell proliferation (*PDGFRA*, *CUL3*, *KIT*, *NACC1*, *RARA*, *BIRC6*, *GAB2*, *CACUL1*, *RUNX2*, *GNAI2*) were subjected to expression analysis (Supplementary Figure S1) and survival analysis to determine their relationship with prognostic value in CC patients. The expression data were analyzed using the KM estimator (Figure 3), and it was found that while high levels of NACC1 (Figure 3a) and CACUL1 (Figure 3b) were associated with better prognosis, high expression of RUNX2 was associated with significantly lower survival (HR = 2.17, 95% CI = 1.14–4.14, *p* < 0.05) (Figure 3c). On the other hand, low levels of miR-218-5p also correlated with lower survival among patients with CC (Figure 1d).

### 2.4. RUNX2 Participates in the Positive Regulation of Cell Proliferation in CC

**Figure 4:**
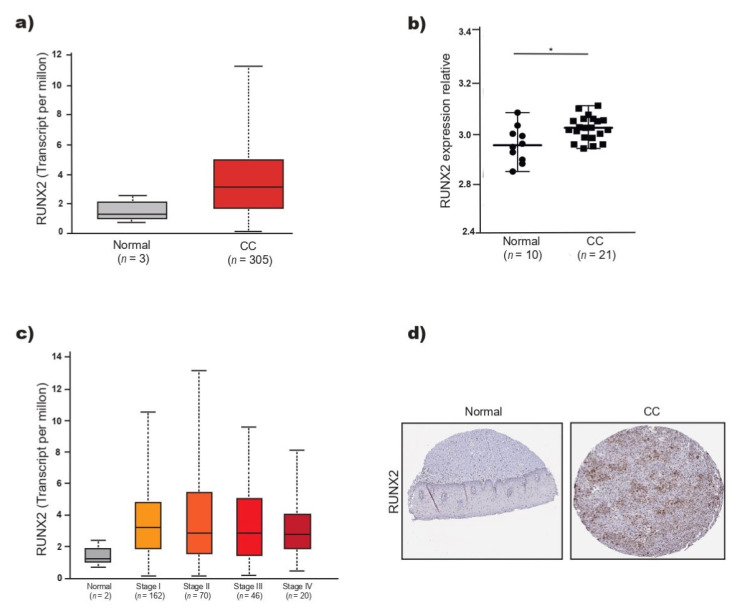
RUNX2 expression in CC. (**a**) RUNX2 expression in CC and normal tissue (TCGA data). (**b**) RUNX*2* expression from the GSE7803 dataset, that includes CC tissues (invasive squamous cell carcinoma, positive for intermediate–risk or high-risk oncogenic HPV) and normal tissues (squamous cervical epithelium). (**c**) RUNX2 expression by stage of cervical tumor. (**d**) RUNX2 expression detected by immunohistochemistry in normal and CC tissues (image available online https://www.proteinatlas.org/ENSG00000124813–RUNX2/pathology/cervical+cancer#img accessed on 25 April 2022). * *p <* 0.05.

The level of RUNX2 expression was analyzed using data from 305 patients with CC and three normal tissues recorded in TCGA. The level of RUNX2 mRNA tended to increase in CC but was not significantly different from that observed in normal tissue (Figure 4a). Analysis of the expression of RUNX2 in the GSE7803 dataset showed that the level of RUNX2 was significantly higher in CC than in normal cervical tissue (Figure 4b) but did not differ among CC stages (Figure 4c). RUNX2 expression is increased in the I–IV stages of CC (Figure 4c). Additionally, the expression of RUNX2 was analyzed in tissue samples stained by immunohistochemistry and stored in the HPA, and greater expression of RUNX2 was observed in CC than in normal tissue (image available online https://www.proteinatlas.org/ENSG00000124813-RUNX2/pathology/cervical+cancer#img, accessed on 25 April 2022) (Figure 4d).

### 2.5. Overexpression of miR-218-5p Decreases the Expression of RUNX2 in C-33A and CaSki Cells

**Figure 5:**
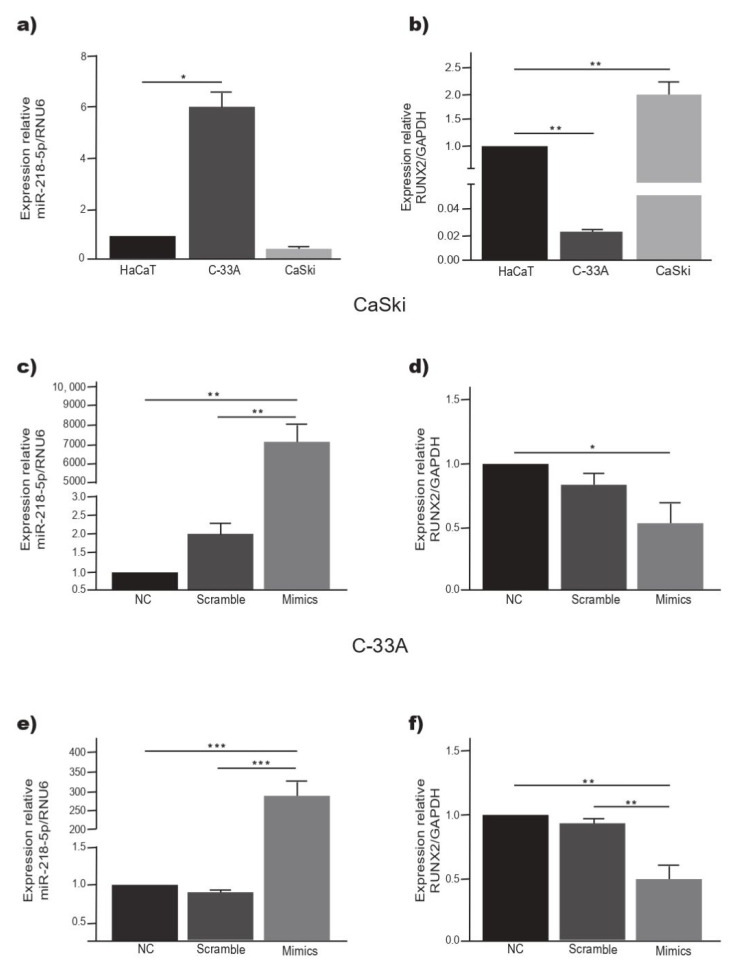
miR-218-5p overexpression is related to a decrease in the RUNX2 level. (**a**) Basal expression of miR-218-5p in HaCaT, C-33A and CaSki cells. (**b**) Basal level of RUNX2 in HaCaT, C-33A and CaSki cells. (**c**) miR-218-5p level and (**d**) RUNX2 expression in CaSki cells transfected with miR-218-5p mimic. (**e**) miR-218-5p expression and (**f**) RUNX2 level in C-33A cells transfect with miR-218-5p mimic. * *p <* 0.05, ** *p* < 0.01, *** *p* < 0.001.

The expression of miR-218-5p was significantly higher in C-33A cells than in HaCaT and CaSki cells (*p* < 0.05) (Figure 1a). On the other hand, RUNX2 was overexpressed in CaSki cells and significantly decreased in C-33A cells (*p* < 0.05) with respect to the HaCaT cell line (Figure 1b). To observe the relationship between the level of miR-218-5p and the expression of RUNX2, C-33A and CaSki cells were transiently transfected with miR-218-5p mimetic or with miR-control. The expression of miR-218-5p increased significantly in both cell lines (Figure 5c,e). In C-33A and CaSki cells overexpressing miR-218-5p, the expression of RUNX2 mRNA was significantly decreased (Figure 5d,f). These data suggest that miR-218-5p regulates the expression of RUNX2 mRNA in C-33A and CaSki cells.

## 3. Discussion

miRNAs are small, highly conserved, noncoding RNAs that regulate the expression of a large number of genes that influence the development and progression of tumors.

Numerous data document alterations in the level of miRNAs that transcriptionally and post-transcriptionally regulate the expression of genes involved in the modulation of cancer hallmarks. Deregulated miRNAs are grouped into tumor suppressors or oncogenic miRNAs (oncomicroRNAs). In CC, the deregulation of the expression of various miRNAs is a molecular mechanism that promotes tumor progression, and it is known that tumor suppressor oncomicroRNAs and miRNAs modulate processes such as proliferation, EMT, migration, invasion and metastasis. In the cervical tissue of women with CC and with HSIL or grade CIN II/III the expression of miR-218-5p is significantly decreased compared to that LSIL or CINI and that in normal tissue [10,11,12,13,14,15,16,17]; therefore, in CC, miR-218-5p is categorized as a tumor suppressor miRNA [12]. In CC cell lines, it has been found that miR-218-5p regulates the expression of oncogenes such as *IDO1*, which contributes to the regulation of apoptosis [12], and *DCUN1D1* [20], which participates in the modulation of invasion; however, there are few studies on the expression of miR-218-5p in women with CC, and there are fewer studies on the relationship between the level of miR-218-5p and its validated or probable target genes in patients.

In agreement with the published data, in this work, it was found that the expression of miR-218-5p is decreased in CC tissue samples. The level of miR-218-5p decreases as the FIGO tumor stage increases and is significantly lower in CC than in noncancerous cervical tissue. The lack of significant differences in the miR-218-5p level between FIGO stages of the tumor may be due to differences in the evolution time of CC, the presence or absence of HPV in the samples, the viral genotype in HPV+ tumors, and the genetic diversity of the women studied, in addition to the fact that the expression of miRNAs is tissue- and time-specific. The decrease in the expression of this miRNA has been associated with an increase in tumor size, the degree of cellular differentiation, metastasis and invasion [11,13,18], all of which are processes that contribute to cancer progression. In addition, it was found that in patients with CC and low miR-218-5p expression, the probability of OS was significantly lower (*p* = 0.002) than in women with cancer and high expression of this miRNA [11,12,19]. This behavior can be explained according to results found in CC cell lines, where it has been shown that some miR-218-5p target genes participate in the regulation of proliferation, apoptosis, EMT, migration, invasion or metastasis. The dissemination of CC to the liver, bone or lung and resistance to treatment are frequent causes of death among women with CC, and it is likely that miR-218-5p target genes act in synergy to promote metastasis and resistance to apoptosis, all of which are processes that contribute to shortening the life of the patient with CC. Thus, the expression level of miR-218-5p can be useful as a prognostic predictor of OS in CC.

The prediction of potential miR-218-5p targets in TargetScan, miRTarBase and miRDB resulted in 118 candidate genes, 17 (14%) of which were previously validated as miR-218-5p targets. This result agrees with the statement that each miRNA regulates more than 100 mRNAs [34]. The enrichment analysis of KEGG pathways revealed that 15% (18/118) of the candidate genes are involved in cancer pathways or in the PI3K–AKT signaling pathway. The results of the GO annotation indicated that 86 genes were enriched in the function of protein binding. Among the enriched biological processes, the positive regulation of cell proliferation ranked first, and the positive regulation of migration ranked fifth. On the one hand, these results correlate with each other; on the other hand, it has been reported that protein binding can stimulate cell proliferation [35]. Additionally, in cell lines, it has been observed that miR-218-5p is linked to the regulation of proliferation and migration through the function of specific target genes [10,13,19]. The results obtained in this study open up the possibility of expanding our knowledge about the variety of genes that can be deregulated in CC and about how miR-218-5p regulates signaling pathways and cellular processes related to cancer. *PDGFRA*, *CUL3*, *KIT*, *NACC1 RARA*, *BIRC6*, *GAB2*, *CACUL1*, *GNAI2* and *RUNX2* are among the target genes of miR-218-5p that participate in the positive regulation of cell proliferation, a biological process most enriched in the GO annotation. PDGFRA and RUNX2 were validated as direct targets of miR-218-5p: PDGFRA in T98G cells (glioblastoma multiforme) [36] and RUNX2 in HEK293T cells (human embryonic kidney) [37], in the K-1 and TPC-1 cells lines (papillary thyroid cancer) [32], in SKOV3 cells (ovarian cancer) [38], in ARPE-19 cells (retinal pigment epithelium) [39], and in the A549 cell line (lung adenocarcinoma) [40]. These data strengthen the results of the prediction analysis performed in this study.

Based on the TCGA data, the relationship between the expression levels of PDGFRA, CUL3, KIT, NACC1, RARA, BIRC6, GAB2, CACUL1, GNAI2 and RUNX2 and the OS of patients with CC was analyzed. The KM estimator indicated that only the expression levels of CACUL1, NACC1 and RUNX2 have prognostic value; however, high expression of CACUL1 and NACC1 is associated with a higher OS in patients with CC, and high levels of RUNX2 are significantly correlated with lower OS than patients with low expression of RUNX2. While RUNX2 expression is increased in tissues with CC, miR-218-5p expression is decreased; however, the RUNX2 level does not increase with the tumor stage, while miR-218-5p expression decreases. It is likely that this difference is due to the fact that the RUNX2 mRNA is regulated by other miRNAs, in addition to miR-218-5p. RUNX2 is a direct target of miR-218-5p [40], and is also regulated by miR-466 [41,42]. miR-466 is overexpressed in CC tissue [43]. On the other hand, it is feasible that in CC, the level of RUNX2 is regulated by other transcriptional and posttranscriptional mechanisms, in addition to miR-218-5p. RUNX2 is a TF that regulates proliferation and the cell cycle [23] and is overexpressed in tumors arising from epithelial tissues such as the breast [25], pancreas [26], prostate [27] and lung [28], among others. In breast cancer, high expression of RUNX2 is associated with lower survival and is positively correlated with the risk of bone metastasis [44]. Han et al. [32] reported that 2.5% of 22,972 women with CC developed bone metastasis, and it is likely that RUNX2 is involved in this process.

In C-33A cells, the expression of miR-218-5p is higher, and the level of RUNX2 mRNA is significantly lower than that in nontumorous HaCaT cells; conversely, in the CaSki cell line, the expression of RUNX2 is higher, and the level of miR-218-5p is lower than that in HaCaT cells. The level of miR-218-5p was similar to that found by other authors [12]. The differences in the expression of miR-218-5p and RUNX2 between cancer cell lines may be related to the specific characteristics of each type. On the one hand, it has been reported that the E6 oncoprotein of HPV16 regulates the expression of miR-218-5p and that CaSki cells are HPV16-positive [9,16,45]. Additionally, E6 of HPV 16 positively regulates DNA methyltransferases such as DNMT1 and induces the hypermethylation of miRNA promoters such as miR-375 in CC cell lines [46]. On the other hand, in the tissue of patients with CC and HPV16 infection, it has been found that the miR-218-5p promoter is methylated [47]. These data explain, at least in part, the low level of miR-218-5p in CaSki cells. In addition, CaSki cells are derived from the metastatic site, and the results of RUNX2 expression agree with those reported in the metastatic cell lines MDA-MB-231, Sum49 and Sum59 of breast cancer, which have high expression of RUNX2 [48,49].

In contrast, in nonmetastatic MCF-7, Bt474 and skb3 cells, the expression of this TF is lower [48], as was found in C-33A cells in this study. The increase in RUNX2 expression in metastatic cell lines is related to the participation of this TF in the positive regulation of metastasis [29,49]. These results suggest that FT RUNX2 may be a prognostic factor related to the survival of patients with CC.

The exogenous overexpression of miR-218-5p in C-33A and CaSki cells induced a significant reduction in the expression of RUNX2. The results suggest that miR-218-5p participates in the negative regulation of RUNX2 in CC cell lines; however, more studies are needed to determine the participation of RUNX2 in the progression of CC. In breast cancer, the positive regulation of metastasis through the ABL/RUNX2/MMP13 axis has been reported. ABL is a tyrosine kinase that directly binds, phosphorylates and activates RUNX2 through its SH2 domain, while activated RUNX2 promotes the expression of MMP13, which favors MDA-MB-231 cell invasion [50]. RUNX2 regulates EMT in breast cancer through the activation of the Wnt pathway [48], and in prostate cancer, hyperactivation of AKT by RUNX2 has been observed [51]. On the other hand, RUNX2 positively regulates the YAP signaling pathway in gastric cancer cells [52], and the activation of YAP1 promotes CC [53]. It is likely that RUNX2 also activates the YAP1 pathway in CC.

In addition, RUNX2 regulates pathways such as PI3K/AKT [32,54,55], NF-κB [56], Wnt/β-catenin [57] and TGF-β [58]. Interestingly, in papillary thyroid cancer, the positive regulation of miR-218-5p and the inhibition of RUNX2 induce the inactivation of the PTEN/PI3K/AKT pathway [32]; it is likely that this event occurs in CC. There are very few reported studies on the miR-218-5p and RUNX2 in CC. The results of this study suggest that miR-218-5p regulates RUNX2 and that the levels of both molecules can be useful as prognostic markers; however, the molecular mechanisms through which the miR-218-5p/RUNX2 axis contributes to CC progression are unknown.

It is important to recognize the limitations of this study. First, for the analysis of miR-218-5p and RUNX2 expression, data recorded in TCGA were used, and it was not possible to include HPV infection in the KM estimator. This can cause differences in the results, as observed in CaSki cells with respect to C-33A. Second, the sample size is small. Undoubtedly, studies are required that include a greater number of data points that allow the grouping of CC patients based on HPV type in the tumor. Third, the prediction analysis of miR-218-5p targets was performed with bioinformatics tools that include few confirmed targets; therefore, it is likely that among the predicted genes, there are several unconfirmed miR-218-5p targets.

## 4. Materials and Methods

### 4.1. Prediction of miR-218-5p Target Genes

The prediction of miR-218-5p target genes was performed in TargetScan v.8.0 [59,60], miRTarBase v.7.0 [61] and miRDB v.6.0 [62,63]. The targets that were predicted by the three tools, as indicated by the Venn diagrams (http://bioinformatics.psb.ugent.be/webtools/Venn/, accessed on 20 April 2022), and miR-218-5p target genes were considered.

### 4.2. GO Enrichment and KEGG Pathway Analysis of the miR-218-5p Target Genes

miR-218-5p target genes were subjected to functional enrichment analysis (Gene Ontology, GO) and signaling pathway analysis in the Kyoto Encyclopaedia of Genes and Genomes (KEGG) through the Database for Annotation, Visualization and Integrated Discovery v.6.8 (DAVID) [64,65] to understand the molecular functions, cellular components, biological processes and pathways in which these target genes participate. The pathways were organized based on their enrichment scores. A *p* value < 0.05 was the criterion for significant enrichment.

### 4.3. Expression Analysis in CC Samples from the TCGA, GEO and HPA Databases

The gene expression data and diagnoses of the patients were obtained from The Cancer Genome Atlas (TCGA) and were used to generate box plots that show the expression levels of miR-218-5p and RUNX2 in CC biopsies in different stages and in normal cervical tissue. The databases UALCAN [66], Gene Expression Profiling Interactive Analysis (GEPIA), [67] and Gene Expression Omnibus (GEO), [68,69] were used, and the latter includes the datasets GSE86100 and GSE7803. The dataset GSE86100 (GPL19730 Agilent-046064 Unrestricted_Human_miRNA_V19.0_Microarray) [70] is composed of 6 samples of squamous cell carcinoma positive for HPV16 and 6 samples of healthy cervical tissues, without HPV infection. The dataset GSE7803 (GPL96 Affymetrix Human Genome U133A Array) [71] includes 10 samples of normal squamous cervical epithelium and 21 samples of invasive squamous cell carcinoma positive for HR-HPV or intermediate risk. Expression data were grouped by tumor stage based on the classification of the International Federation of Gynecology and Obstetrics (FIGO). The expression of RUNX2 in nontumor cervical tissue and CC was illustrated with images of tissues stained by immunohistochemistry obtained from the Human Protein Atlas (HPA) (image available online https://www.proteinatlas.org/ENSG00000124813-RUNX2/pathology/cervical+cancer#img, accessed on 25 April 2022).

### 4.4. Survival Analysis

The survival data of patients diagnosed with CC in different stages, available in TCGA, were analyzed to determine the correlation between the level of gene expression and overall survival (OS). The median value of expression was considered as the cutoff value. The tissues with superior levels to cutoff value were included in the group of high expression, and the tissues with levels below were grouped of low expression. The Kaplan–Meier curve was constructed for the low or high expression groups for miR-218-5p (*n* = 307, low expression = 88, high expression 219), RUNX2 (*n* = 304, low expression = 76, high expression = 228) and miR-218-5p target genes that were involved in cell proliferation. The gene expression results of squamous cell carcinoma samples were included in the study. The analysis was performed in the Kaplan–Meier (KM)–plotter program [72,73]. The hazard ratio (HR) and 95% confidence interval were calculated, and a *p* value < 0.05 was indicated statistical significance.

### 4.5. Cell Culture

The CC cell lines C-33A (ATCC^®^ HTB-31ᵀᴹ) and CaSki (ATCC^®^ CLR-1550ᵀᴹ) were purchased from the American Type Culture Collection (ATCC, Manassas, VA, USA). The HaCaT cell line was donated by the National Cancer Institute. The HaCaT cells are transformed cells of epithelial origin, similar to normal keratinocytes. C-33A and CaSki cells were cultured in Dulbecco’s modified Eagle’s medium (DMEM) high in glucose (Invitrogen, Carlsbad, CA, USA). HaCaT cells were cultured in DMEM F12 medium (Caisson, Smithfield, WA, USA). The culture media were supplemented with 10% fetal bovine serum (FBS) (Gibco, Grand Island, NE, USA) and 1% penicillin and streptomycin (Invitrogen, Waltham, MA, USA). All cell lines were incubated at 37 °C with 5% CO_2_.

### 4.6. Cell Transfection

C-33A and CaSki cells in log phase were resuspended and incubated in 6-well plates at a density of 4 × 10^5^ cells/well. After 24 h, at approximately 80% confluence, the cells were transfected with 50 nM hsa-miR-218-5p mimic (assay ID: MC10328) or miR negative control (Scramble), both obtained from Ambion (Austin, TX, USA). Transfection was performed using Lipofectamine 2000 (Invitrogen, Carlsbad, CA, USA) following the protocol indicated by the manufacturer.

### 4.7. RNA Extraction and qRT–PCR

The total RNA of the transfected and untransfected HaCaT, C-33A and CaSki cells was extracted with TRIzol (Invitrogen, Carlsbad, CA, USA) according to the manufacturer’s instructions. The concentration and purity of the total RNA were evaluated in a UV–Vis NanoDrop 2000c spectrophotometer (Thermo Fisher Scientific, Waltham, MA, USA). For the analysis of miR-218-5p expression, 5 ng of total RNA was reverse transcribed for miRNAs with the TaqMan^®^ MicroRNA Reverse Transcription kit (Applied Biosystems, Vilnius, Lithuania) following the manufacturer’s instructions. The expression of miR-218-5p (Assay ID: 000521) was determined with the TaqMan microRNA assay kit (Applied Biosystems, Vilnius, Lithuania). For the expression of RUNX2 (Assay ID: Hs00231692_m1), the TaqMan RNA–to–CT 1-Step Kit (Applied Biosystems, Vilnius, Lithuania) was used. qRT-PCR were run on Prism 7500 equipment (Applied Biosystems, Foster City, CA, USA). The relative expression of miR-218-5p and RUNX2 was determined with the 2^−ΔΔCt^ method [74] using RNU6 (Assay ID: 001973) or GAPDH (Assay ID: Hs99999905_m1) as the normalization control. All tests were performed in triplicate.

### 4.8. Statistical Analysis

The TCGA data were analyzed with the Welch *t*–test, which estimates the magnitude of the differences in the expression levels of a specific gene between normal tissues and primary tumors or tumor subgroups. The differences in the variables that were evaluated between two experimental groups were calculated with the GraphPad Prism program (version 5.01; GraphPad Software, Inc., San Diego, CA, USA) with Student’s *t*-test. Data are shown as the mean ± SD. A *p* value < 0.05 indicated statistical significance.

## 5. Conclusions

In summary, low miR-218-5p expression in the tissues of CC patients is associated with lower OS, and in C-33A and CaSki cells, the level of miR-218-5p is related to the characteristics of each cell type. Bioinformatics analysis revealed 118 miR-218-5p target genes, and a significant number of them positively regulate proliferation, migration or protein binding. RUNX2 is among the miR-218-5p targets and participates in the positive modulation of cell proliferation. The data recorded in TCGA indicate that RUNX2 overexpression is related to a worse CC prognosis. Overall, low levels of miR-218-5p and high expression of RUNX2 are associated with lower OS in CC. Both molecules can be useful as prognostic markers. In C-33A and CaSki cells, miR-218-5p overexpression decreases RUNX2 mRNA expression, and this dynamic supports the hypothesis that RUNX2 is regulated by miR-218-5p in CC.

## Figures and Tables

**Table 1:** miR-218-5p target genes in overlap in TargetScan, miRTarbase and miRDB databases.

118 Genes Predicted to Be Targeted by miR-218-5p			
*ACTN1*	*GLCE*	*NFATC3*	*SH3GL1*
*ANKRD27*	***GNAI2***	*NRAS*	*SH3KBP1*
*ANKRD52*	*GNAS*	*NUFIP2*	*SHMT1*
*ARPP19*	*GNB1*	*OTUD7B*	*SHOC2*
*ATXN1*	*HECTD2*	*PAIP2*	*SIK2*
*BCL11B*	*HNRNPA3*	***PDGFRA***	*SLAIN2*
*BCL9*	*HP1BP3*	*PHC3*	*SLC38A1*
***BIRC6***	*ITM2C*	*PKP4*	*SLC45A3*
*BMI1*	*KIAA1522*	*PPP1CC*	*SNX4*
***CACUL1***	*KIF21B*	*PPP2R2A*	*SOST*
*CCDC6*	*KIRREL3*	*PRKG1*	*SPAG9*
*CDH2*	***KIT***	*PTP4A1*	*SREK1*
*CNTNAP2*	*KLF12*	*PUM2*	*SRSF10*
*COL1A1*	*KLF9*	*PURB*	*STAM2*
***CUL3***	*KLHL13*	*PRLR*	*THOC2*
*DAZAP1*	*KMT2A*	***RARA***	*TOB1*
*DCBLD2*	*LARP4B*	*RBM18*	*TPD52*
*DCP2*	*LASP1*	*RET*	*TTYH3*
*DDX6*	*LGR4*	*RHOQ*	*UBE2H*
*DOCK9*	*LMNB1*	*RICTOR*	*UBN2*
*DUSP18*	*LRIG1*	*RNF219*	*USP34*
*ELL2*	*LRRFIP1*	*RNF38*	*VAT1*
*FAM214A*	*LYPD6B*	*ROBO1*	*VOPP1*
*FAM217B*	*MAFG*	*RPP25*	*WASF3*
*FBN2*	*MARCKS*	*RPS6KA3*	*WIPF2*
*FBXO41*	*MBNL1*	*RPS6KB1*	*ZFX*
*FYCO1*	*MBNL2*	***RUNX2***	*ZFYVE26*
***GAB2***	*MITF*	*SATB2*	*ZNF609*
*GFPT1*	*NAA15*	*SEPHS1*	
*GJA1*	***NACC1***	*SETBP1*	

Bold letters: miR-218-5p target genes involved in positive regulation of cell proliferation.

## Data Availability

Publicly available datasets were analyzed in this study. This data can be found online: http://ualcan.path.uab.edu/; https://kmplot.com/analysis/index.php?p=background (accessed on 25 April 2022); https://www.ncbi.nlm.nih.gov/gds; http://gepia.cancer-pku.cn/ (accessed on 2 June 2022).

## Supplementary Materials

The following supporting information can be downloaded at: www.mdpi.com/article/10.3390/ijms23136993/s1.
