# Supplementary material for: miR-218-5p/RUNX2 Axis Positively Regulates Proliferation and Is Associated with Poor Prognosis in Cervical Cancer
Source: Int J Mol Sci. 2022 Jun 23;23(13):6993. doi: 10.3390/ijms23136993 (PMC9267020; doi:10.3390/ijms23136993)
Supplement: Supplementary file 1 [file ijms-23-06993-s001.zip › supplementary Figure S1.pdf]

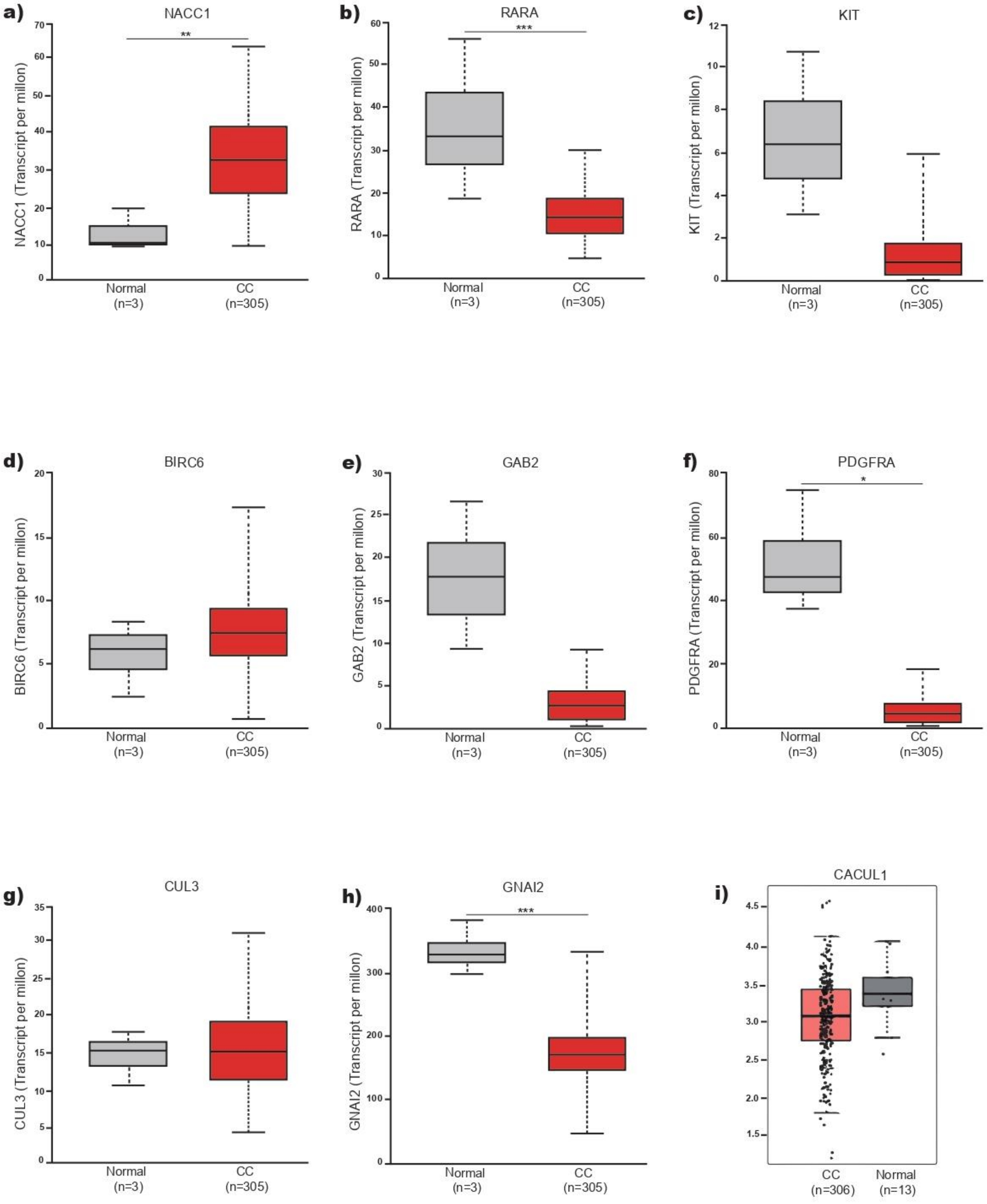

Supplementary Figure S1. Expression of miR-218-5p target genes. (**a-h**) Expression of target genes of miR-218-5p involved in the regulation of cellular proliferation in CC tissue. (**a**) NACC1, (**b**) RARA, (**c**) KIT, (**d**) BIRC6, (**e**) GAB2, (**f**) PDGFRA, (**g**) CUL3, (**h**) GNAI2 (data from UALCAN database). (**i**) CACUL1 (data from GEPIA). * $p<0.05$, ** $p<0.01$, *** $p<0.001$.
